# miR-25-3p, Positively Regulated by Transcription Factor AP-2α, Regulates the Metabolism of C2C12 Cells by Targeting *Akt1*

**DOI:** 10.3390/ijms19030773

**Published:** 2018-03-08

**Authors:** Feng Zhang, Kun Chen, Hu Tao, Tingting Kang, Qi Xiong, Qianhui Zeng, Yang Liu, Siwen Jiang, Mingxin Chen

**Affiliations:** 1 Hubei Key Laboratory of Animal Embryo Engineering and Molecular Breeding, Institute of Animal Husbandry and Veterinary, Hubei Academy of Agricultural Sciences, Wuhan 430064, China; zhangfeng0130@163.com (F.Z.); taohu00@gmail.com (H.T.); phenixxq@163.com (Q.X.); liuyang430209@163.com (Y.L.); 2 Key Laboratory of Swine Genetics and Breeding of the Agricultural Ministry and Key Laboratory of Agricultural Animal Genetics, Breeding and Reproduction of the Ministry of Education, College of Animal Science and Technology, Huazhong Agricultural University, Wuhan 430070, China; kunchen1989@163.com (K.C.); 13163228175@126.com (T.K.); zengqianhui.hzau.cn@webmail.hzau.edu.cn (Q.Z.)

**Keywords:** mouse, miR-25-3p, *Akt1*, AP-2α, promoter, cell metabolism

## Abstract

miR-25, a member of the miR-106b-25 cluster, has been reported as playing an important role in many biological processes by numerous studies, while the role of miR-25 in metabolism and its transcriptional regulation mechanism remain unclear. In this study, gain-of-function and loss-of-function assays demonstrated that miR-25-3p positively regulated the metabolism of C2C12 cells by attenuating phosphoinositide 3-kinase (*PI3K*) gene expression and triglyceride (TG) content, and enhancing the content of adenosine triphosphate (ATP) and reactive oxygen species (ROS). Furthermore, the results from bioinformatics analysis, dual luciferase assay, site-directed mutagenesis, qRT-PCR, and Western blotting demonstrated that miR-25-3p directly targeted the AKT serine/threonine kinase 1 (*Akt1*) 3′ untranslated region (3′UTR). The core promoter of miR-25-3p was identified, and the transcription factor activator protein-2α (AP-2α) significantly increased the expression of mature miR-25-3p by binding to its core promoter in vivo, as indicated by the chromatin immunoprecipitation (ChIP) assay, and AP-2α binding also downregulated the expression of *Akt1*. Taken together, our findings suggest that miR-25-3p, positively regulated by the transcription factor AP-2α, enhances C2C12 cell metabolism by targeting the *Akt1* gene.

## 1. Introduction

MicroRNAs (miRNAs) are endogenous, small (~22 nucleotides), and single-stranded noncoding RNAs. The role of different miRNAs in biological systems is well established. They are generally regarded as negative regulators of gene expression, as they bind to the 3′ untranslated region (3′UTR) of messengerRNAs (mRNAs), leading to mRNA degradation and/or suppression of mRNA translation [1,2,3]. Currently, thousands of miRNAs have been identified as participating in a number of biological processes, such as cellular growth, proliferation, development, and metabolism [4].

Based on Solexa sequencing, the expression of microRNA-25 (miR-25) was higher in the longissimus dorsi muscle of Large White pigs (a lean type) than in those of Tongcheng pigs (a Chinese indigenous fatty pig). Because skeletal muscle plays a vital role in whole-body metabolism [5], we speculated that miR-25 could play a regulatory role in metabolism.

Previous studies have reported that miR-25 plays an important role in many biological processes. The expression of miR-25-3p was significantly increased in the plasma of thyroid papillary carcinoma, as compared with patients with benign tumors or healthy individuals [6]. miR-25 expression was higher in ovarian epithelial tissue, gastric cancer, lung adenocarcinoma, and many other tumors, and miR-25 expression levels were also closely related to tumor stage and lymph node metastasis [7,8,9,10]. Inhibition of miR-25 markedly improved cardiac contractility in the failing heart [11]. miR-25 could protect cardiomyocytes against oxidative damage by downregulating the mitochondrial calcium uniporter (MCU) [12]. Variations in miR-25 expression influenced the severity of diabetic kidney disease [13]. However, to our knowledge, the role of miR-25 in metabolism has not been reported, and its transcriptional regulatory mechanism is not clear.

Thus, in this study, we first investigated whether miR-25 was involved in metabolism by gain-of-function and loss-of-function assays. Then, the target gene of miR-25, AKT serine/threonine kinase 1 (*Akt1*), which is related to metabolism, was predicted and verified using bioinformatics software and experiments. Finally, the core promoter of miR-25 was identified, and the binding of the transcription factor activator protein-2α (AP-2α) to the core promoter was shown to promote the transcriptional activity of miR-25 and downregulate *Akt1* expression.

## 2. Results

### 2.1. miR-25 Is Highly Conserved in Mammals

**Figure 1:**
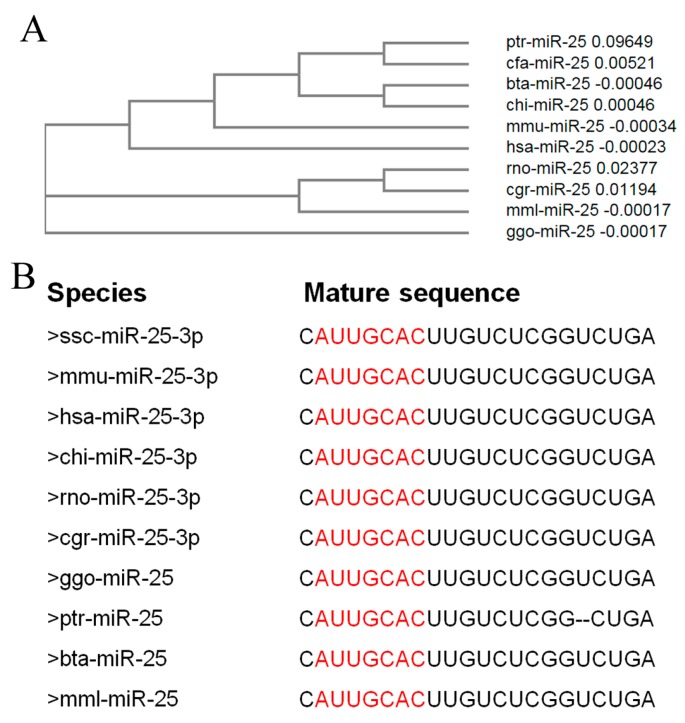
miR-25 is highly conserved in mammals. (**A**) The phylogenetic tree of pre-miRNA of miR-25. pre-miRNA sequences were obtained from NCBI. (**B**) The mature sequences of miR-25 in selected species. These mature sequences were obtained from miRBase. Seed regions are highlighted in red. ssc, sus scrofa; mmu, mus musculus; hsa, homo sapiens; chi, capra hircus; rno, rattus norvegicus; cgr, cricetulus griseus; ggo, gorilla gorilla; ptr, pan troglodytes; bta, bos taurus; mml, macaca mulatta; cfa, canis lupus familiaris.

Clustal Omega (Available online: https://www.ebi.ac.uk/Tools/msa/clustalo/) [14] was used to build the phylogenetic tree of pre-miRNA of miR-25. The results show that compared with other species selected in this study, the genetic relationship between mice and humans, cattle and goats, and gorillas and rhesus monkeys is closer (Figure 1A). The mature sequences of miR-25 are highly conserved in mammals, including pigs, mice, humans, goats, rats, hamsters, gorillas, chimpanzees, cattle, and rhesus monkeys. The “seed” sequences of miR-25 are identical, although there is a base deletion at the end of the chimpanzee sequence (ptr) (Figure 1B). 

### 2.2. Effects of miR-25 on the Metabolism of C2C12 Cells

**Figure 2:**
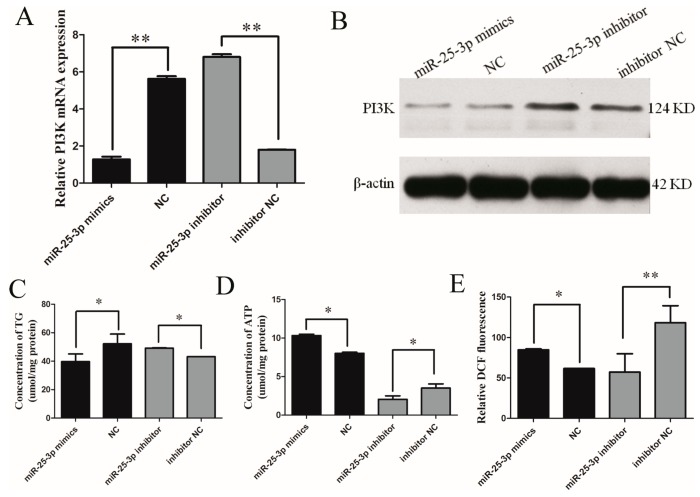
The effect of miR-25 on the metabolism of C2C12 cells. miR-25-3p mimics/NC or inhibitors/NC were respectively transfected into growing C2C12 cells. After 48 h, PI3K expression was detected by qRT-PCR (**A**) and Western blotting (**B**). After 24–48 h transfection, the levels of triglyceride (TG) (**C**), ATP (**D**), and reactive oxygen species (ROS) (**E**) were measured with commercial kits. The fluorescence of DCF represents the content of ROS. NC = negative control (miR-239b-5p of caenorhabditis elegans). β-actin served as the loading control. Data were presented as means ± SD (*n* ≥ 3); * *p* < 0.05; ** *p* < 0.01.

To investigate the role of miR-25-3p in metabolism, miR-25-3p mimics/negative control (NC) or inhibitors/NC were respectively transfected into growing C2C12 cells (mouse muscle myoblasts). The abundance of miR-25-3p was detected, which was ~3300-fold (*p* < 0.01) higher as compared with another microRNA (Figure S1). The mRNA and protein expression levels of the metabolism-related gene *PI3K* were repressed by miR-25-3p overexpression, while the levels of *PI3K* were upregulated in the inhibitor group, as compared with the negative controls (Figure 2A,B).

In addition, the overexpression of miR-25-3p decreased levels of triglyceride (TG), whereas the knockdown of miR-25-3p increased them (Figure 2C). Conversely, the overexpression of miR-25-3p increased ATP and ROS levels, and the knockdown of miR-25-3p decreased their levels (Figure 2D,E).These data indicate that miR-25-3p plays a role in metabolism.

### 2.3. miR-25-3p Directly Targets Akt1

**Figure 3:**
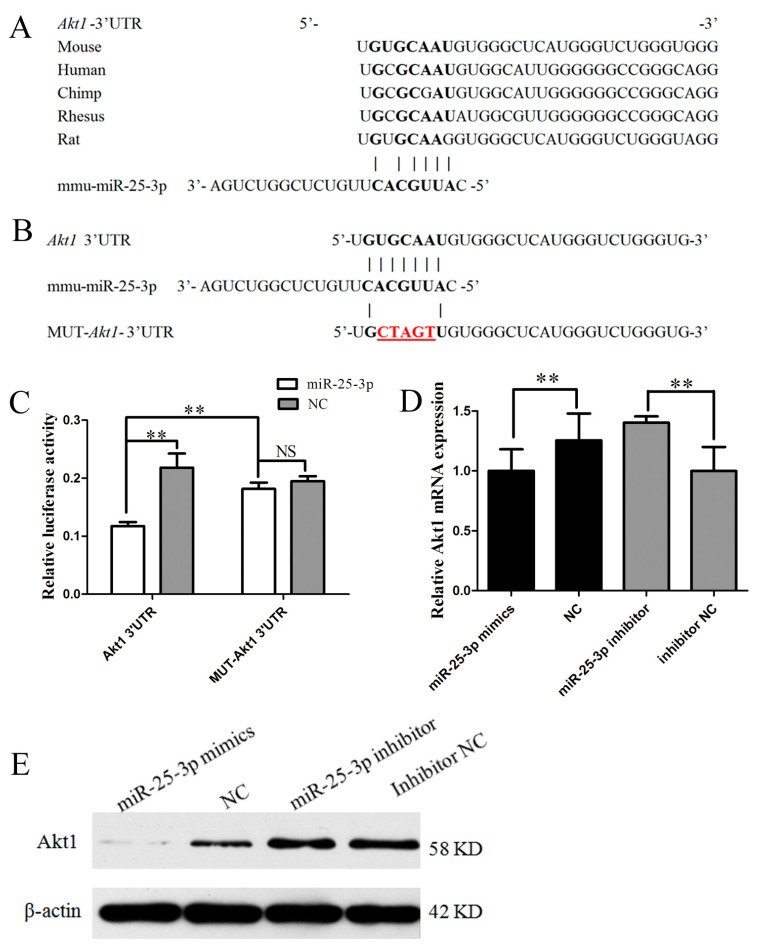
miR-25-3p directly targets the 3′UTR of *Akt1*. (**A**) The sequences of miR-25-3p target elements in the *Akt1* 3′UTR were relatively conserved in many mammals. These sequences were obtained from TargetScan. (**B**) Site-directed mutagenesis of the miR-25-3p target site in the *Akt1* 3′UTR; mutated bases shown in red. (**C**) Dual luciferase reporter assay. The *Akt1* 3′UTR/mutant plasmid was cotransfected with miR-25-3p mimics/NC, respectively, into growing C2C12 cells; dual luciferase activities were measured from cell lysates (24 h after transfection). miR-25-3p mimics/NC or inhibitors/NC were respectively transfected into growing C2C12 cells. After 48 h, *Akt1* expression was detected by qRT-PCR (**D**) and Western blotting (**E**). NC = negative control (miR-239b-5p of caenorhabditis elegans). β-actin served as the loading control. Data were presented as means ± SD (*n* ≥ 3). ** *p* < 0.01; NS, not significant.

To explore the molecular mechanism of miR-25-3p effects on metabolism, the possible targets for miR-25-3p were predicted using TargetScan, and a putative binding site for miR-25-3p was predicted in the 3′UTR of *Akt1* mRNA. miR-25-3p targeting elements in the *Akt1*-3′UTR were relatively conserved in many mammals, including mice, humans, chimpanzees, rhesus monkeys, and rats (Figure 3A).

To validate whether miR-25-3p directly targets *Akt1*, a luciferase reporter containing a 250 bp fragment from the *Akt1* 3′UTR was tested in vitro. Additionally, we generated a mutated version of the above mentioned reporter, in which five nucleotides of the predicted binding site were changed in order to abolish the putative interaction between miR-25-3p and *Akt1* mRNA (Figure 3B). The *Akt1* 3′UTR and mutant luciferase plasmid were cotransfected with mimics or NC into growing C2C12 cells. Twenty-four hours after transfection, analyses of luciferase activity revealed that miR-25-3p mimics significantly decreased the luciferase activity of the wild reporter plasmid as compared with NC, while there was no significant effect on the mutant plasmids (Figure 3C). These results revealed that miR-25-3p directly targets the 3′UTR of *Akt1* in vitro.

To directly test the validity of the putative target, we transfected miR-25-3p mimics and miR-25-3p inhibitors into growing C2C12 cells. We found that the overexpression of miR-25-3p repressed *Akt1* expression, as measured by qRT-PCR (*p* < 0.01) and Western blotting, whereas the knockdown of miR-25-3p derepressed it (Figure 3D,E). These results demonstrate that *Akt1* was a target of miR-25-3p. 

### 2.4. Identification and Characterization of the Mouse miR-25-3p Promoter

**Figure 4:**
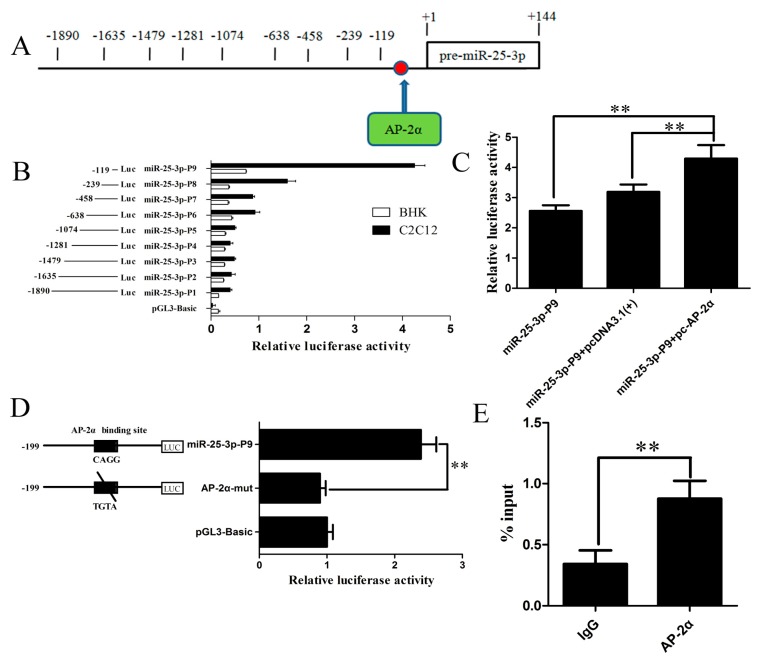
Transcription factor AP-2α binds to the miR-25-3p promoter region. (**A**) Schematic diagram of the AP-2α binding site (arrow, red dot) in the miR-25-3p promoter. The first nucleotide of pre-miR-25-3p was assigned as +1, and the other nucleotides were numbered relative to it. (**B**) A series of progressive deletion mutants were transfected into growing BHK and C2C12 cells, and the promoter activities were analyzed by dual luciferase activity assay. (**C**) miR-25-3p-P9 reporter constructs were cotransfected with pc-AP-2α into growing C2C12 cells. Dual luciferase activity was measured 24 h after transfection. Overexpression of AP-2α upregulated miR-25-3p promoter luciferase activity. pcDNA-3.1(+) was used as a control. (**D**) Site-directed mutagenesis of the AP-2α binding site (CAGG into TGTA) in the miR-25-3p promoter region resulted in the miR-25-3p-P9 luciferase activity being reduced. Data were expressed as the ratio of relative activity, normalized to pRL-TK, and presented as means ± SD (*n* ≥ 3). (**E**) Binding of AP-2α to the miR-25-3p promoter region was analyzed by chromatin immunoprecipitation (ChIP). DNA isolated from immunoprecipitated materials was amplified using qRT-PCR. Normal mouse IgG was used as the negative control. Data were normalized by total chromatin (input) and presented as means ± SD (*n* = 3); ** *p* < 0.01.

To further identify the promoter region and regulatory elements of mouse miR-25-3p, we used luciferase assays to analyze a series of deletions in the potential promoter region, as predicted by neural network promoter prediction (NNPP) online software (Figure 4A).The plasmids containing the various lengths of the miR-25-3p promoter were transiently transfected into growing BHK and C2C12 cells. Analyses of luciferase activity revealed that miR-25-3p-P9 (−119/+144) showed the greatest transcriptional activity, and the longer fragment showed lower transcriptional activity (Figure 4B), indicating that the region from −1870 to −119 contains one or more *cis*-acting elements that can repress miR-25-3p expression. The result demonstrates that this 263 bp-long sequence was the core promoter of mouse miR-25-3p.

### 2.5. The Transcription Factor AP-2α Binds to the Core Promoter of Mouse miR-25-3p

To further search the transcription factors that bind to the core promoter of mouse miR-25-3p, AliBaba 2.1 and Genomatix software programs were utilized to analyze the putative transcription factors. As shown in Figure S2, AP-2α was found to be able to bind to the core promoter of mouse miR-25-3p. To examine whether AP-2α influences the activity of the mouse miR-25-3p promoter, an AP-2α overexpression plasmid (pc-AP-2α) was generated and cotransfected with the miR-25-3p-P9 plasmid into growing C2C12 cells. Twenty-four hours after transfection, analyses of luciferase activity showed that pc-AP-2α significantly increased miR-25-3p promoter transcriptional activity (Figure 4C). 

To determine the functional importance of the AP-2α binding site, we mutated the AP-2α binding site at −109 to −102, by using the wild-type miR-25-3p-P9 plasmid as the template. The mutant was constructed and transfected into growing C2C12 cells. As shown in Figure 4D, the luciferase activity of the mutant was significantly decreased as compared with the wild-type miR-25-3p-P9 construct. These results indicated that transcription factor AP-2α may induce transcriptional activity by directly binding to the core promoter of mouse miR-25-3p.

To further verify whether transcription factor AP-2α binds to the core promoter of mouse miR-25-3p, ChIP was performed in growing C2C12 cells. Chromatin was immunoprecipitated using the AP-2α antibody, and PCR amplification was performed, using the DNA fragment of the expected size as a template. The ChIP-Q-PCR assay showed that AP-2α interacted with the miR-25-3p promoter within the binding site (Figure 4E). These results confirmed that the transcription factor AP-2αis capable of binding to the AP-2α binding site in the mouse miR-25-3p promoter region, and induces miR-25-3p transcription.

### 2.6. AP-2α Regulates miR-25-3p and Akt1 Expression

**Figure 5:**
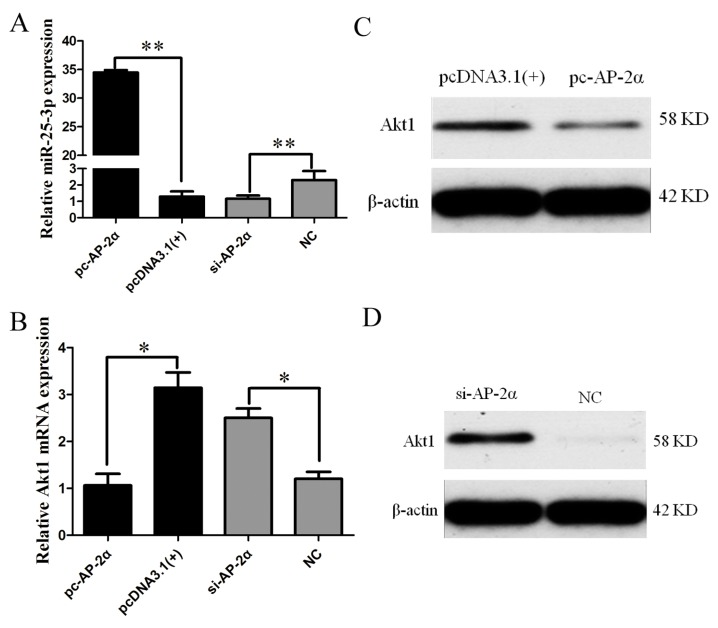
The effects of AP-2α on the expression of miR-25-3p and *Akt1*. The eukaryotic expression plasmid pc-AP-2α or si-AP-2α was transfected into growing C2C12 cells. After 48 h, the expression of miR-25-3p and *Akt1* was detected by qRT-PCR and Western blotting. (**A**) The expression of miR-25-3p was detected by qRT-PCR. (**B**) The mRNA expression of *Akt1* was detected by qRT-PCR. Data were presented as means ± SD (*n* = 3); * *p* < 0.05; ** *p*< 0.01. (**C**) The protein expression of Akt1 was detected by Western blotting after pc-AP-2α transfection. (**D**) The protein expression of Akt1 was detected by Western blotting after si-AP-2α transfection. β-actin served as the loading control.

Because *Akt1* was identified as a direct target of miR-25-3p, and the transcription factor AP-2α could upregulate miR-25-3p transcription, the effect of AP-2α on *Akt1* expression was further appraised by the overexpression or knockdown of AP-2α in growing C2C12 cells. As AP-2α mRNA expression was significantly decreased by doublestranded short interfering AP-2α RNA ( si-AP-2α-1) and si-AP-2α-2, and the inhibitory effect of si-AP-2α-2 was greater than that of si-AP-2α-1 (Figure S3), si-AP-2α-2 was chosen for subsequent experiments. pc-AP-2α or si-AP-2α was transfected into growing C2C12 cells, respectively. Fourty-eight hours after transfection, RNA and protein were isolated. The overexpression of AP-2α significantly increased miR-25-3p expression, while the knockdown of AP-2α resulted in the significant suppression of miR-25-3p expression (Figure 5A). Conversely, the mRNA and protein expression of Akt1 were significantly suppressed by AP-2α overexpression, and were increased by si-AP-2α (Figure 5B–D). These results indicate that AP-2α activated mature miR-25 expression, and downregulated the expression of *Akt1*.

## 3. Discussion

Increasing evidence shows that miR-25, a member of the miR-106b-25 cluster, is involved in many biological processes. For instance, miR-25 inhibits human gastric adenocarcinoma cell apoptosis [15], promotes glioblastoma cell proliferation and invasion [16], and regulates human ovarian cancer apoptosis [17]. The miR-106b-25 cluster regulates adult neural stem/progenitor cell proliferation, migration, and differentiation [18,19]. miR-25 plays an important role in heart disease [11,12] and diabetic kidney disease [13]. In addition, numerous studies have demonstrated that miRNAs are implicated in metabolism [20,21,22,23]. However, miR-25 has not been functionally related to metabolism until now.

In this study, miR-25 was identified as a novel regulator of metabolism. The gain-of-function and loss-of-function assays showed that miR-25-3p inhibited the expression of *PI3K* and reduced levels of triglyceride (TG), while levels of ATP and ROS were increased. PI3K has been implicated in insulin-regulated glucose metabolism [24], and PI3K signaling has a role in many cellular processes, such as metabolic control, immunity, and cardiovascular homeostasis [25,26,27]. It is well-known that triglycerides (TG) are a component of lipids, and participate in lipid metabolism. ATP is the most direct source of energy in an organism, and takes part in many metabolic processes. ROS, a class of single electron radicals of oxygen, comprise superoxide anions (O_2_^−^), hydrogen peroxide (H_2_O_2_), and hydroxyl radicals (**^·^**OH) [28], and are closely related to adipogenesis and myogenesis [28,29,30,31]. These data indicate that miR-25-3p indeed participates in metabolism in mice.

To further understand the molecular mechanism by which miR-25-3p regulates metabolism, we searched for potential target genes of miR-25-3p via TargetScan. Fortunately, the 3′UTR of *Akt1* contained a 7 nucleotides perfect match site complementary to the miR-25-3p seed region (Figure 3B). The serine-threonine kinase ATK, also known as protein kinase B (PKB), is an important effector for PI3K signaling as initiated by numerous growth factors and hormones [32]. *Akt* can control glucose uptake by regulating GLUT4 in cells, thereby reducing blood sugar and promoting glycogen synthesis [32,33,34]. *Akt* usually promotes glycogen synthase kinase-3 alpha (GSK3α) phosphorylation and inhibits its activity [35], and then activates glycogen synthesis [36]. A previous study has demonstrated that overexpression of miR-25-3p downregulates *Akt* expression and inactivates Akt phosphorylation in the tongue squamous cell carcinoma cell line Tca8113 [37]. Consequently, we deduced that the role of miR-25-3p in metabolism may arise from its inhibition of *Akt1*. First, the dual luciferase reporter assay demonstrated that *Akt1* was a direct target of miR-25-3p, shown by the steady decrease luciferase activity of the pmirGLO-Akt1-wt vector; but not the mutant form (Figure 3C). Meanwhile, qRT-PCR and Western blotting results showed that the expression of *Akt1* was inhibited by the miR-25-3p mimics, and that this inhibition was reversed by the miR-25-3p inhibitors (Figure 3D,E). These results suggested that the effect of miR-25-3p in metabolism was due, at least in part, to the suppression of *Akt1*.

An increasing number of studies have shown that transcription factors are capable of binding to miRNA promoter elements and modulating miRNA transcription [38,39,40]. Therefore, we analyzed the transcriptional mechanism of miR-25-3p in this study. Nine fragments of 5′-flanking sequences of mouse miR-25-3p were isolated. Subsequently, a series of experiments, including dual luciferase, site-directed mutagenesis, and ChIP assays, confirmed that AP-2α bound to the miR-25-3p promoter region and promoted its transcription activity (Figure 4). Moreover, qRT-PCR and Western blotting results showed that overexpression of AP-2α resulted in the upregulation of miR-25-3p and downregulation of *Akt1*, and that the knockdown of AP-2α reversed these results (Figure 5).

The AP-2 family of transcription factors consists of five members, in humans and mice: AP-2α, AP-2β, AP-2γ, AP-2δ, and AP-2ε; which play important roles in several cellular processes, such as apoptosis, migration, and differentiation [41,42]. AP-2α was first identified by its ability to bind to the enhancer regions of SV40 and human metallothionein IIA [43]. Subsequently, numerous studies have demonstrated that AP-2α can regulate gene expression. For instance, AP-2α binding to the *C/EBPα* promoter results in decreased *C/EBPα* expression [44], and AP-2α can bind to the *TACE* promoter and decrease its expression in dendritic cells [45]. Furthermore, Qiao et al. [46] reported that there was an AP-2α binding site in the *DEK* core promoter, and overexpression of AP-2α upregulated *DEK* expression. In this study, we identified that AP-2α binds to the miR-25-3p promoter region and promotes its transcription activity.

**Figure 6:**
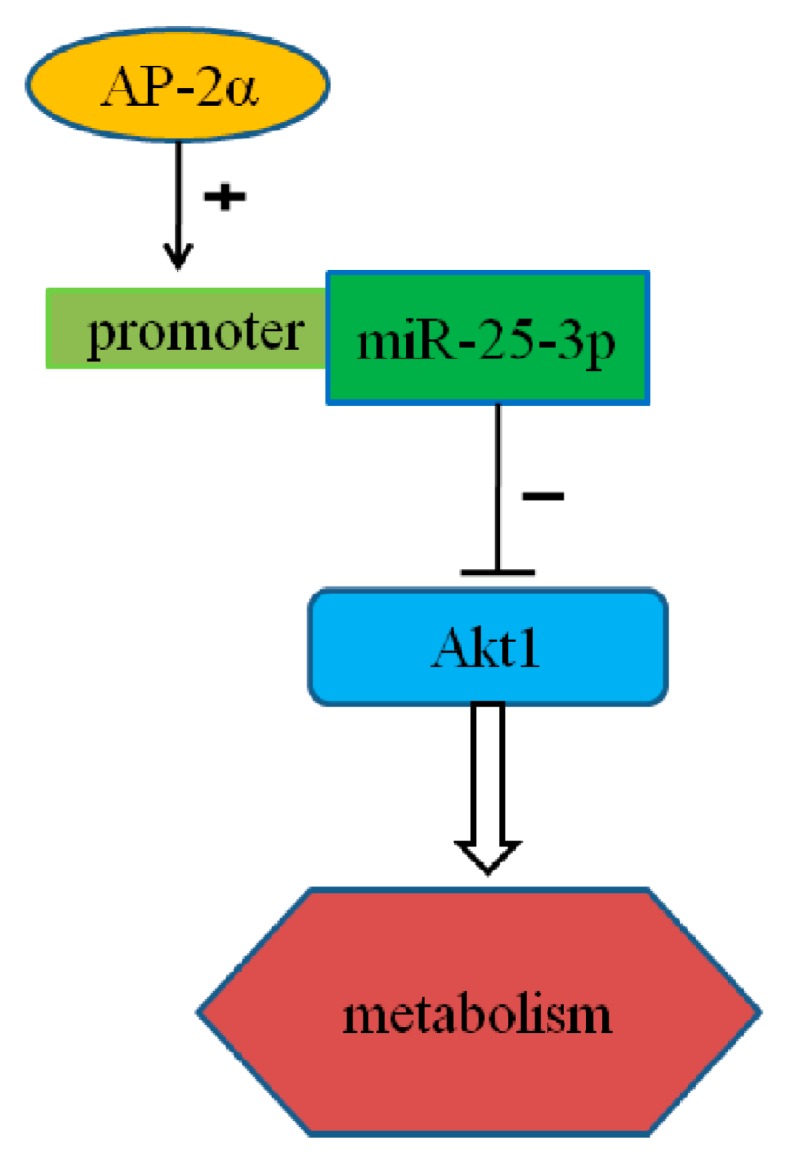
Representation of the proposed mechanism. miR-25-3p is regulated by transcription factor AP-2α, and contributes to C2C12 metabolism by targeting *Akt1*. The arrow-head and “+” represent activation while the blunt-head and “−“ represent suppression.

In conclusion, our results demonstrate that miR-25-3p acts as a positive regulator of the metabolism of growing C2C12 cells, by affecting *Akt1* gene expression through directly binding to its 3′UTR. Moreover, the transcription factor AP-2α is able to bind to the core promoter of mouse miR-25-3p, activating mature miR-25 expression and downregulating the expression of *Akt1* (Figure 6).

## 4. Materials and Methods

### 4.1. miRNA, Small RNA Oligonucleotide Synthesis, and Plasmid Construction

The miR-25-3p oligonucleotides (miR-25-3p mimics, NC, miR-25-3p inhibitors, and inhibitor-NC) and double-stranded short interfering RNAs (siRNAs) targeting AP-2α were designed and synthesized by RiboBio (Guangzhou, China).The oligonucleotides are listed in Table S1.

To construct the AP-2α overexpression vector pc-AP-2α, the AP-2α coding sequence (1314 bp) was amplified from mouse C2C12 cells cDNA using the following primers: forward: 5′-CCCAAGCTTGCCACCATGCTTTGGAAACTGACGGA-3′; reverse: 5′-CCGCTCGAGTCACTTTCTGTGTTTCTCTT-3′. The PCR product was subcloned into the *Hind*III/*Xho*I sites of the pcDNA3.1(+) vector (Invitrogen, Carlsbad, CA, USA).

The potential target site of miR-25-3p, localized in the 3′UTR of *Akt1* mRNA, was predicted by TargetScan (Available online: http://www.targetscan.org/) [47]. The *Akt1* 3′UTR was amplified from C2C12 cell cDNA and inserted into the *Pme*I/*Xho*I sites of the pmirGLO vector (Promega, Madison, WI, USA). Point mutations in the seed region of the predicted miR-25-3p sites within the 3′UTR of *Akt1* were generated using overlap-extension PCR [48]. The corresponding primers are listed in Table S2.

The potential promoter regions of miR-25-3p was predicted by using the neural network promoter prediction (NNPP) software (Available online: http://www.fruitfly.org/seq_tools/promoter.html) [49]. Nine miR-25-3p promoter deletion fragments were amplified from the mouse genome via PCR with the primers listed in Table S3.The nine purified PCR products were ligated into the *Kpn*I/*Hind*III sites of the pGL3-Basic vector (Promega). AliBaba2.1 (Available online: http://www.gene-regulation.com/) [50] and MatInspector (Available online: http://www.genomatix.de/online_help/help_matinspector/matinspector_help.html) [49] were used to predict the potential transcription factor binding sites. The AP-2α transcription factor binding sites of the miR-25-3p promoter region were also mutated by overlap-extension PCR. The primers are provided in Table S3.

### 4.2. Cell Culture and Luciferase Reporter Assays

C2C12 (mouse muscle myoblast) and BHK (baby hamster kidney) cells were cultured in DMEM (Gibco, Gaithersburg, MD, USA) containing 10% fetal bovine serum (FBS) (Gibco) at 5% CO_2_ and 37 °C.

For luciferase reporter assays, growing C2C12 or BHK cells were seeded in 48-well plates. After 12–16 h, the plated cells were transfected with a recombinant plasmid using Lipofectamine 2000 (Invitrogen). To verify the miR-25-3p targeting *Akt1* 3′UTR, 1 μL miR-25-3p mimics/NC was cotransfected with 0.1 μg *Akt1* 3′UTR/mutant plasmid into C2C12 cells. For the miR-25-3p promoter luciferase reporter assay, 0.4 μg pGL3-Basic or recombinant plasmids and 20 ng pRL-TK vector were transfected. For cotransfection luciferase assays, each well contained 0.2 μg pGL3-(Basic, miR-25-3p-P9 and AP-2α-mut), 20 ng pRL-TK, and 0.2 μg pc-AP-2α. Empty pcDNA-3.1(+) cotransfected with pGL3-(Basic, miR-25-3p-P9 and AP-2α-mut) was used as the control. After 24 h of incubation, luciferase activity was measured using a PerkinElmer 2030 Multilabel Reader (PerkinElmer, Norwalk, CT, USA).

### 4.3. Triglyceride Content, ATP, and Reactive Oxygen Species (ROS) Assays

For detecting the concentrations of triglyceride (TG), ATP, and ROS, growing C2C12 cells were seeded in 24-well plates the day before transfection. miR-25-3p mimic, NC, miR-25-3p inhibitor, and inhibitor-NC were transfected into confluent (~80%) cells, respectively, at a concentration of 12 nM with Lipofectamine 2000 (Invitrogen). After 24–48 h, the concentrations of TG and ATP in the lysates of cells were measured with commercial kits (Applygen (Beijing, China) and Beyotime (Shanghai, China), respectively) following the manufacturer’s instructions, and normalized to the protein content (μmol/mg protein) using the BCA assay kit (Thermo Scientific, Waltham, MA, USA). ROS were measured using the reactive oxygen species assay kit (Beyotime) following the manufacturer’s protocol.

### 4.4. Chromatin Immunoprecipitation (ChIP)

ChIP assays were performed to assess the binding of endogenous AP-2α to the miR-25-3p promoter in C2C12 cells using the EZ-ChIP™ Kit (Millipore, Boston, MA, USA), following a previously described method [49]. Precleared chromatin was incubated with the AP-2α antibody (Santa Cruz Biotechnology, Dallas, TX, USA) or normal mouse IgG (Millipore) antibodies (control) overnight at 4 °C. Purified DNA from the samples and the input controls were analyzed for the presence of miR-25-3p promoter sequences containing putative AP-2α response elements using qPCR. The primers used here are listed in Table S4.

### 4.5. RNA Isolation and qRT-PCR

For quantifying the mRNA expression of genes, growing C2C12 cells were seeded in 6-well plates. miR-25-3p mimic, NC, miR-25-3p inhibitor, inhibitor-NC, si-AP-2α, and NC were transfected into confluent (~80%) cells, respectively, at a concentration of 50 nM with Lipofectamine 2000 (Invitrogen). After 48 h, total RNA was isolated using a HP Total RNA Kit (Omega, Norcross, GA, USA) according to the manufacturer’s protocol. The cDNA was synthesized using a PrimeScript™RT reagent Kit with gDNA Eraser (Takara, Osaka, Japan) according to the manufacturer’s protocol. The qRT-PCR was performed in triplicate with iQSYBR green Supermix (Bio-Rad, Hercules, CA, USA) in a LightCycler 480 Realtime PCR machine (Roche, Basel, Switzerland). The mRNA levels of target genes were reported relative to those of the house keeping gene β-actin by using the 2^−ΔΔ^*^C^*^t^ method. The qRT-PCR primers are listed in Table S5.

### 4.6. Protein Isolation and Western Blotting

For detecting the protein expression of PI3K and Akt1, growing C2C12 cells were seeded in6-well plates. miR-25-3p mimic, NC, miR-25-3p inhibitor, inhibitor-NC, si-AP-2α, and NC were transfected into confluent (~80%) cells, respectively, at a concentration of 50 nM with Lipofectamine 2000 (Invitrogen). After 48 h, total protein was isolated using RIPA Lysis Buffer (Beyotime). The cells were washed briefly with cold phosphate-buffered saline (PBS), 150 μL RIPA Lysis Buffer (containing 1 mM PMSF) was added, incubated for 1 min at room temperature, and then centrifuged at 12,000× *g* for 5 min. The supernatant extract was used for Western blot analysis. 

Western blot analysis was performed to analyze the expression levels of Akt1 (Affinity Biosciences, Cincinnati, OH, USA) andPI3K (Abclonal, Wuhan, China) according to the methods of Huang et al. [47]. β-actin (Santa Cruz Biotechnology) served as the loading control.

### 4.7. Statistical Analysis

All the results are presented as the means ± SD. Student’s *t*-test was used for statistical comparisons. A *p* value of < 0.05 was considered to be statistically significant. ** *p* < 0.01; * *p* < 0.05; NS, not significant.

## Supplementary Materials

The following are available online at http://www.mdpi.com/1422-0067/19/3/773/s1.
