# Supplementary material for: miR-25-3p, Positively Regulated by Transcription Factor AP-2α, Regulates the Metabolism of C2C12 Cells by Targeting Akt1
Source: Int J Mol Sci. 2018 Mar 8;19(3):773. doi: 10.3390/ijms19030773 (PMC5877634; doi:10.3390/ijms19030773)
Supplement: Supplementary file 1 [file ijms-19-00773-s001.pdf]

International Journal of
*Molecular Sciences*

MDPI

## Supplementary Materials

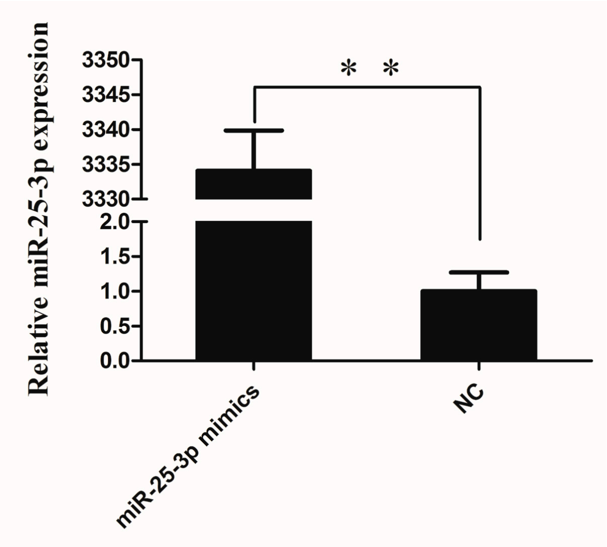


**Figure S1.** The mRNA expression of miR-25-3p was detected by qRT-PCR. NC, negative control (miR-239b-5p of caenorhabditis elegans). Data were presented as means ± SD ($n$ = 3), ** $p < 0.01$.

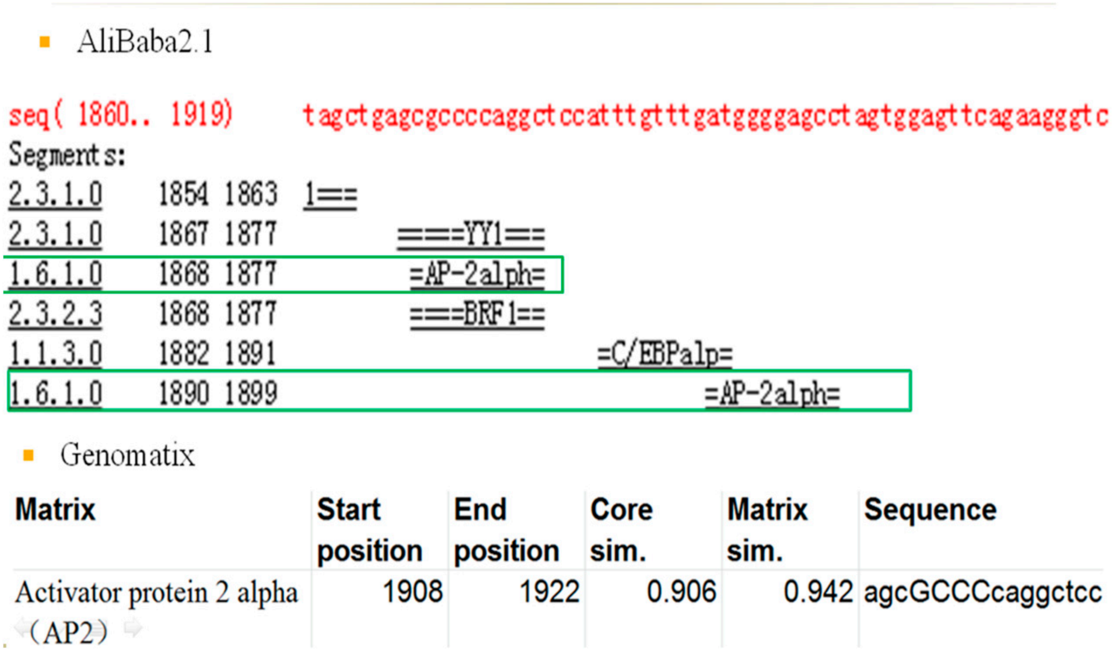

▪ AliBaba2.1

seq( 1860.. 1919) tagctgagcgccccaggctccatttgtttgatggggagcctagtggagttcagaagggtc

Segments:

| | | | |
|---|---|---|---|
| 2.3.1.0 | 1854 | 1863 | 1== |
| 2.3.1.0 | 1867 | 1877 | ===YY1== |
| 1.6.1.0 | 1868 | 1877 | =AP-2alph= |
| 2.3.2.3 | 1868 | 1877 | ===BRF1== |
| 1.1.3.0 | 1882 | 1891 | =C/EBPalp= |
| 1.6.1.0 | 1890 | 1899 | =AP-2alph= |

▪ Genomatix

| Matrix | Start position | End position | Core sim. | Matrix sim. | Sequence |
|---|---|---|---|---|---|
| Activator protein 2 alpha (AP2) | 1908 | 1922 | 0.906 | 0.942 | agcGCCCcaggctcc |

**Figure S2.** Transcription factor AP-2α binding sites in the core promoter of mouse miR-25-3p were predicted by AliBaba 2.1 and Genomatix softwares programs.

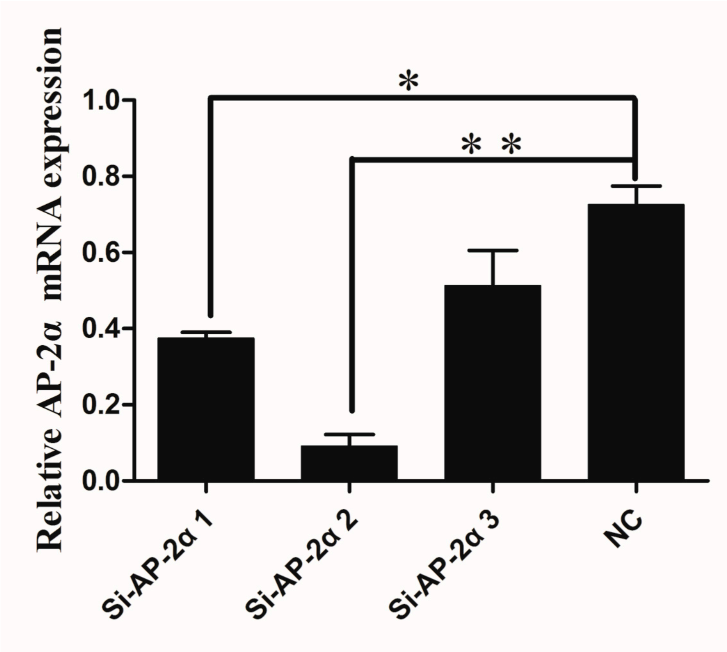


**Figure S3.** The mRNA expression of AP-2*α* was detected by qRT-PCR. Data were presented as means ± SD ($n$ = 3), * $p < 0.05$; ** $p < 0.01$.

**Table S1.** Oligonucleotide sequences of miR-25-3p and AP-2*α* siRNAs.

| Name | Sequence (5′-3′) |
|---|---|
| miR-25-3p mimics | CAUUGCACUUGUCUCGGUCUGA |
| | UCAGACCGAGACAAGUGCAAUG |
| miR-25-3p NC | UUUGUACUACACAAAAGUACUG |
| | CAGUACUUUUGUGUAGUACAAA |
| miR-25-3p inhibitor | UCAGACCGAGACAAGUGCAAUG |
| miR-25-3p inhibitor-NC | CAGUACUUUUGUGUAGUACAA |
| siAP-2*α*-1 | F: GGAGAGCGAAGUCUAAGAA*TT* |
| | R: UUCUUAGACUUCGCUCUCC*TT* |
| siAP-2*α*-2 | F: CCAAAGCAGUAGCAGAAUU*TT* |
| | R: AAUUCUGCUACUGCUUUGG*TT* |
| siAP-2*α*-3 | F: GCAUCCUUCGAGCAAUAAA*TT* |
| | R: UUUAUUGCUCGAAGGAUGC*TT* |

Note: Italics bases represent protective bases.

**Table S2.** Primers for amplification of *Akt1* 3′ UTR and its mutated fragment.

| Name | Primer sequence (5′-3′) |
|---|---|
| Akt1 | F:*GG*GTTTAAACGCCAGGTCAGGAGGAAAACTA |
| | R:*CCG*CTCGAGCTTCTGGTGCCTTCTTGCTG |
| | Mut-F:GACGCGACCATGCTAGTTGTGGGCTCATG |
| | Mut-R:CATGAGCCCACAACTAGCATGGTCGCGTC |

Note: Italics, protective bases; underlined, enzyme loci; shadow, mutation sites

**Table S3.** Primers for amplification of miR-25-3p promoters and AP-2*α* transcription factor binding sites mutated fragment.

| Name | Primer sequence (5′-3′) | Size (bp) | Tm (°C) |
|---|---|---|---|
| miR-25-3p-P1 | F: *GG*GGTACCACTGACTTTAGAGGGCGGTG | 2034 | 61 |
| | R: *CC*AAGCTTTGTCAGACCGAGACAAGTGC | | |
| miR-25-3p-P2 | F: *GG*GGTACCGCTTTCTCGATTCGACCTCCT | 1779 | 60 |
| | R: *CC*AAGCTTTGTCAGACCGAGACAAGTGC | | |
| miR-25-3p-P3 | F:*GG*GGTACCGCAGACACCTTAAACACCTTTCC | 1623 | 60 |

| | | | |
|---|---|---|---|
| | R: *CC*AAGCTTTGTCAGACCGAGACAAGTGC | | |
| miR-25-3p-P4 | F: *GG*GGTACCCATCACCTATGTCCACCAGCA<br>R: *CC*AAGCTTTGTCAGACCGAGACAAGTGC | 1425 | 60 |
| miR-25-3p-P5 | F: *GG*GGTACCCCTGGGTCTCTCTGTAGCCA<br>R: *CC*AAGCTTTGTCAGACCGAGACAAGTGC | 1218 | 60 |
| miR-25-3p-P6 | F: *GG*GGTACCTTCCCGCTTTCCCATGAACC<br>R: *CC*AAGCTTTGTCAGACCGAGACAAGTGC | 782 | 60 |
| miR-25-3p-P7 | F: *GG*GGTACCTCTCCGACTTTCCACTGCTC<br>R: *CC*AAGCTTTGTCAGACCGAGACAAGTGC | 602 | 59 |
| miR-25-3p-P8 | F: *GG*GGTACCAGGCTGCTTGCTGCTTGAAT<br>R: *CC*AAGCTTTGTCAGACCGAGACAAGTGC | 383 | 59 |
| miR-25-3p-P9 | F: *GG*GGTACCCCCCCAGGACACAACCTCTG<br>R: *CC*AAGCTTTGTCAGACCGAGACAAGTGC | 263 | 59 |
| AP-2α-mut | F: TAGCTGAGCGCCCTGTACTCCATTTGTTTGA<br>R:TCAAACAAATGGAGTACAGGGCGCTCAGCTA | | |

Note: Italics, protective bases; underlined, enzyme loci.

**Table S4.** Primers for ChIP assays.

| Name | Primer sequence (5′-3′) | Size (bp) | Tm (℃) |
|---|---|---|---|
| ChIP-AP-2α | F: TGCTGAGCTAGCACTTCCC<br>R: CTGTCCTGTGAGGGAGACCA | 132 | 60 |

**Table S5.** Primers for qRT-PCR.

| Primer | Primer sequence（5′-3′） | Size(bp) | Tm (℃) |
|---|---|---|---|
| miR-25-3p Loop | CTCAACTGGTGTCGTGGAGTCGGCAATTCAGTT GAGTCAGACCG | 66 | 60 |
| miR-25-3p | F: **CTGGTAGG**CATTGCACTTGTCT<br>R: TCAACTGGTGTCGTGGAG | | |
| U6 Loop | CTCAACTGGTGTCGTGGAGTCGGCAATTCAGTT GAGAAAAATATGGAACGCT | | |
| U6 | F: **CTGGTAGG**GTGCTCGCTTCGGCAG<br>R: TCAACTGGTGTCGTGGAG | 150 | 60 |
| Akt1 | F: GATGGAGGCCAGGGTACAAA<br>R: GCAGCGACACCACAAAAATGA | 176 | 59 |
| PI3K | F: GAAATCTCCTGGGATGTGTCGT<br>R: ATCTGGTGGCTCTCGGAGTAA | 223 | 59 |
| β-actin | F: CAAGAGAGGTATCCTGACCCT<br>R: TGATCTGGGTCATCTTTTCAC | 188 | 58 |

Note: red, loop sequences; yellow, universal sequences; bold bases, protective bases.
